# Individual socioeconomic position among the general population and economic downturns-related perceived stress, psychological resilience, and wellbeing in Thailand

**DOI:** 10.3389/fpubh.2026.1855202

**Published:** 2026-06-08

**Authors:** Ratanaporn Awiphan, Chidchanok Ruengorn, Chabaphai Phosuya, Kiatkriangkrai Koyratkoson, Penkarn Kanjanarat, Kednapa Thavorn, Nahathai Wongpakaran, Tinakon Wongpakaran, Surapon Nochaiwong

**Affiliations:** 1 Department of Pharmaceutical Care, Faculty of Pharmacy, Chiang Mai University, Chiang Mai, Thailand; 2 Pharmacoepidemiology and Statistics Research Center (PESRC), Faculty of Pharmacy, Chiang Mai University, Chiang Mai, Thailand; 3 Ottawa Hospital Research Institute, Ottawa Hospital, Ottawa, ON, Canada; 4 Institute of Clinical and Evaluative Sciences, ICES uOttawa, Ottawa, ON, Canada; 5 School of Epidemiology and Public Health, Faculty of Medicine, University of Ottawa, Ottawa, ON, Canada; 6 Department of Psychiatry, Faculty of Medicine, Chiang Mai University, Chiang Mai, Thailand

**Keywords:** inequalities, mental health, psychological resilience, socioeconomic position, stress, wellbeing, social determinants of health

## Abstract

**Background:**

Economic downturns before and during the coronavirus disease 2019 (COVID-19) pandemic have been accompanied by adverse psychosocial outcomes. In Thailand, evidence on how individual-level socioeconomic position (SEP) relates to key psychosocial issues remains limited, and a standardized, simple SEP index is unavailable. We examined the associations between an individual SEP index and adverse psychosocial outcomes among working-aged adults.

**Methods:**

We recruited working-age adults aged 18–60 years using an online public mental health survey conducted in Thailand. The individual SEP index is based on a composite of educational level, personal income, occupational position, and housing overcrowding. The SEP index was classified as very low, low, moderate, and high. Psychosocial issues, including perceived stress, psychological resilience, and wellbeing, were measured using validated instruments. Associations between the SEP index and psychosocial outcomes were analyzed using weighted multivariable Tobit and ordinal logistic hierarchical regression models to account for potential confounders and the ordinal nature of the outcome variables.

**Results:**

Based on 1,992 participants, the sex- and age-adjusted prevalence estimates were 9.8% for the very low, 31.6% for the low, 48.0% for the moderate, and 10.6% for the high SEP index groups, weighted by the national population and Internet use rate. We found a significant association between the SEP index category, particularly among those in the very low group, and the degrees of higher perceived stress (*β* coefficients ranged from 1.96 to 2.94), lower resilience coping (*β* coefficients ranged from −0.74 to −1.64), and wellbeing scores (β coefficients ranged from −5.40 to −11.52). Individuals with a lower SEP index category revealed a significantly higher risk of moderate/high perceived stress [common odds ratio (OR), 3.02; 95% confidence interval (CI), 1.45–6.33; *p* = 0.003], low resilient copers (common OR, 0.44; 95% CI, 0.23–0.86; *p* = 0.016), and very poor wellbeing (common OR, 0.30; 95% CI, 0.16–0.58; *p* < 0.001) compared with the high SEP index group.

**Conclusion:**

A simple individual-level SEP index was associated with perceived stress, psychological resilience, and wellbeing among working-age adults in Thailand during the early COVID-19 period. This index may help identify socioeconomically vulnerable groups for targeted mental health interventions; however, external validation is required before routine use in surveillance.

## Introduction

Before and during the coronavirus disease 2019 (COVID-19) pandemic, a subsequent economic downturn and adverse mental health and psychosocial issues have been observed (1–3). In the early phase of the COVID-19 pandemic in Thailand, although national public health measures and stringent government policies effectively limited the spread of COVID-19, economic sectors and activities experienced downturns. As a result, many Thai workers have experienced financial difficulties due to reduced working hours, irregular salaries or income loss, and temporary or permanent job loss. From a macroeconomic perspective, before the COVID-19 pandemic, Thailand’s economy contracted sharply in 2020, with annual gross domestic product growth declining to −6.2%, alongside an increase in the unemployment rate (4).

Psychosocial outcomes, including perceived stress, psychological resilience, and wellbeing, are key indicators of mental health and quality of life. International evidence consistently shows that lower socioeconomic position (SEP) is associated with higher perceived stress, lower resilience, and poorer subjective wellbeing (5, 6). Studies across diverse cultural contexts further suggest that socioeconomic disadvantages increase vulnerability to stress and reduce coping capacity, thereby undermining wellbeing. However, resilience can buffer stress effects; it is also socially patterned by SEP (7). However, Thailand-based research has rarely examined these relationships using a standardized individual-level SEP index, limiting identification of at-risk groups and the development of targeted public mental health interventions.

Theoretically, macroeconomic downturns tend to aggravate the direct and indirect effects on existing socioeconomic inequalities (8). Prior to the global COVID-19 pandemic, existing studies have suggested that socioeconomic disadvantage was a risk factor for both adverse mental and physical health conditions (9–11). SEP is a multidimensional structural construct that reflects access to resources, prestige, and power. It is distinct from socioeconomic status (SES), which often refers to individual attributes such as income or education, and from area-level deprivation, which captures contextual disadvantages. These distinctions are important for understanding how socioeconomic inequalities shape health and wellbeing, consistent with structural and social stratification frameworks (12, 13). Composite individual-level SEP indices (e.g., the Hollingshead Four-Factor Index, MacArthur Subjective Social Status ladder, and European Socioeconomic Index) capture multiple dimensions of advantage. However, their validity and use in Southeast and East Asian populations remain underexamined. Although socioeconomic gradients in mental health are well documented in Western settings, evidence from Asian contexts, including Thailand, is limited, and cross-cultural qualitative work on aging further highlights the role of digital inclusion and social determinants across Germany, Japan, and Thailand (14).

Regarding adverse mental health outcomes, based on two socioeconomic status indicators (area deprivation and low education), low SEP was associated with increased risk for self-harm, poisoning, psychotic disorders, and disorders due to substance abuse, with adjusted hazard ratios ranging from 1.46 to 1.79 (9). Despite a growing body of evidence regarding the socioeconomic gradient during the COVID-19 pandemic, there are compelling reasons for establishing these findings in the Thai population. First, the majority of the literature uses the SEP based on a complex proxy index, such as neighborhood-level, area- or zone-of-residence, or country-level, defined by multiple deprivations or postal codes (15–18). Second, the effects of socioeconomic inequalities among the general population and their psychological consequences, particularly stress related to economic downturns, psychological resilience, and wellbeing, remain underexplored during the COVID-19 pandemic. Finally, no standardized individual SEP index is available to quantify the socioeconomic gradient in Thailand.

To address these gaps, we examined the association between an individual-level simple SEP index and psychosocial outcomes in a nationwide public mental health survey in Thailand during the early phase of the COVID-19 pandemic. We hypothesized that lower SEP would be associated with higher perceived stress, lower psychological resilience, and poorer wellbeing in a graded or dose–response fashion across SEP levels.

## Methods

### Study design and participants

This open online cross-sectional study was the first round of data collection (Wave I of the survey) conducted from April 21 to May 4, 2020, of the Health Outcomes and Mental Health Care Evaluation Survey Under the Pandemic Situation of COVID-19 (HOME-COVID-19), a nationwide survey conducted through the convenient selection of the population in Thailand (19). The survey was distributed via social media platforms, email lists, and community networks to reach a broad and diverse population while complying with physical distancing policies during the COVID-19 pandemic. Participants voluntarily accessed the survey link and provided informed consent before participation, with no incentive or compensation.

For this study’s purpose, we included only general participants who (i) were Thai citizens, permanent residents, or non-residents with work permits aged ≥18 years and (ii) completed an online questionnaire via the SurveyMonkey^®^ platform (limited one-time participation per unique Internet Protocol address). However, to reflect the public experience of working-age Thai adults, we excluded those aged >60 years and college students from the analysis. Students were excluded because income and occupation typically do not reflect stable SEP, and adults aged >60 years were also excluded because retirement, as is generally the case in Thailand, may decouple occupation and income from working-life SEP. The study is reported in accordance with the Strengthening the Reporting of Observational Studies in Epidemiology (STROBE) Statement (20) and the Checklist for Reporting Results of Internet E-Surveys (CHERRIES) (21).

### Exposure: SEP index measure

We constructed an individual-level SEP index comprising four components: educational attainment, monthly personal income, occupational position, and household overcrowding. Education was grouped into categories aligned with the International Standard Classification of Education 2011 to support transparent cross-level comparisons (22). Occupation was classified as routine/manual, intermediate, or higher professional/managerial to reflect skill-based stratification and employment conditions (23). Personal income was categorized based on the average monthly minimum wage in Thailand into <10,000 baht/month, 10,000–20,000 baht/month, and >20,000 baht/month to create interpretable strata for public health monitoring in Thailand (24). We operationalized household overcrowding based on the Organisation for Economic Co-operation and Development (OECD) definition. Overcrowding was measured as the number of persons aged ≥18 years (or as a couple) per room and classified as not overcrowded (≤1 person or couple/room) or overcrowded (>1 person or couple/room) (25). We then constructed the SEP index based on the equivalized socioeconomic variables described in Table 1. The scores ranged from 0 to 7 points. A higher score indicates higher SEP of the participant.

**Table 1:** Socioeconomic variables.

Variables	Categorized level	Unweighted assigned score
Educational level	Low (no, primary school, or junior high school)	0
	Medium (senior high school, diploma, or high vocational)	1
	High (bachelor’s degree or higher education)	2
Personal income^a^	<10,000 baht/month	0
	10,000–20,000 baht/month	1
	>20,000 baht/month	2
Occupational position^b^	Routine and manual occupations	0
	Intermediate occupations	1
	Higher managerial, administrative, and professional occupations	2
Household overcrowding	Yes: more than one person (or couple) per room	0
	No: one or fewer than one person (or couple) per room	1

SEP index interpretation	SEP score
Very low	0–2 points
Low	3–4 points
Moderate	5–6 points
High	7 points
SEP (possible range)	7 (0–7)

a Based on an average monthly minimum wage of 10,000 baht (USD 261) in 2020 in Thailand.

b In cases where participants reported being unemployed, their last occupational position was considered.

SEP, socioeconomic position.

### Outcomes measures: psychosocial issues

The Thai version of a set of intended questionnaires was used to investigate public responses to economic downturn-related psychosocial issues during the COVID-19 pandemic, as follows:

- Perceived stress: Perceived Stress Scale (PSS) 10-item; this scale was used to measure the perception of stress based on a five-point Likert scale. Responses ranged from 0 (never) to 4 (very often). The total score ranged from 0 to 40, with a higher score indicating a higher degree of stress. Cut-off scores of ≤13, 14–26, and ≥27 points were used to indicate none/minimal, moderate, and high degrees of perceived stress, respectively. A previous study and this sample showed acceptable internal consistency (Cronbach’s *α* = 0.75 to 0.85) (26).
- Psychological resilience: Brief Resilience Coping Scale (BRCS) 4-item; this scale captured tendencies to cope with stress using a five-point Likert scale. Responses ranged from 1 (does not describe me at all) to 5 (describes me very well). The total scores range from 4 to 20, with higher scores indicating greater resilience to cope. The BRCS scores of ≤13, 14–16, and ≥17 were categorized as low, medium, and high resilience copers, respectively. A prior study and this sample demonstrated high internal consistency of the BRCS 4-item (Cronbach’s *α* = 0.84 to 0.86) (27).
- Wellbeing: World Health Organization (WHO) wellbeing index 5-item; this scale was used to measure general health-related personal wellbeing based on a six-point Likert scale. The response options ranged from 0 (no time) to 5 (all times). The total raw score ranges from 0 to 25, and the transformed score is multiplied by 4 to yield a score of 0 to 100. A higher score indicates the best imaginable wellbeing. Scores ≤50 and ≤28 indicate poor and very poor wellbeing, respectively. Prior studies and this sample also demonstrated high internal consistency (Cronbach’s α = 0.87 to 0.92) (28, 29).

### Covariates

Apart from the SEP components (educational level, personal income [baht/month], occupational position, and household overcrowding), covariates were collected to adjust for potential confounding factors. Covariates were selected *a priori* based on their potential associations with socioeconomic position and psychosocial outcomes. Participants self-reported sociodemographic characteristics, including age, sex, marital status, education, occupation-related characteristics, and region of residence. Health-related covariates included self-rated health and the presence of chronic non-communicable diseases (yes/no). Pandemic-related covariates included employment/income changes due to COVID-19 (e.g., reduced working hours, income loss, or job loss; yes/no).

Based on the degree of adjustment for the association between the SEP index and psychosocial issues (perceived stress, psychological resilience, and wellbeing), two adjustment models were added to the unadjusted effect estimates. Considering the participants’ sociodemographic characteristics, Model 1 included age, sexual identity, marital status, religion, region of residence, living status, reimbursement scheme, history of mental illness, and history of non-communicable diseases. Regarding the economic burden related to the COVID-19 pandemic, Model 2 included variables from Model 1 and personal financial status indicators (job loss, income loss, and debt) to examine whether SEP associations changed after accounting for pandemic-related burdens, which may act as downstream mediators. All covariates were entered into multivariable models as categorical variables, except age, which was modeled as continuous data. Multicollinearity testing was performed for the covariates included in the model adjustment.

### Statistical analysis

As per the pre-specified protocol for estimates of a range of mental health and psychosocial issues during the COVID-19 pandemic, 1,310 participants were needed to obtain a type I error probability of 0.05, statistical power of 80%, design effect of 2.0, and response completion rate of 60% (19). All eligible participants who met the study criteria were included in the analysis.

Differences in participant characteristics across the SEP index categories (very low, low, moderate, and high) were evaluated using Fisher’s exact test, analysis of variance, or the Kruskal-Wallis test, as appropriate. Crude associations between SEP components were explored using Spearman’s rank correlation analysis. Owing to the floor and ceiling effects of the psychosocial issues score, the association between the SEP index scores and the mean difference in psychosocial outcomes was investigated using multivariable Tobit hierarchical regression with a series of two adjustment models, as described. To reflect a direction of a worse effect on the PSS score (none/minimal-, moderate-, or high-perceived stress) or better impact on the BRCS score (low-, medium-, or high-resilient copers) and WHO wellbeing score (very poor, poor, or fair/high wellbeing index), we estimated this ratio based on a three-level scale using a multivariable ordinal logistic hierarchical regression analysis to improve interpretability and assess robustness of psychosocial outcomes. The proportional odds assumption was assessed using the Brant test in ordinal logistic regression. In cases where the assumption was violated, less restrictive specifications (e.g., partial proportional odds or generalized ordered logit models) were employed.

The effect estimates for Tobit and ordinal logistic regression analyses were reported as *β* coefficients and common odds ratios (ORs), respectively, corresponding to 95% confidence intervals (95% CIs). To improve representativeness, survey data analyses were weighted according to the national population distribution by age, sex, and regions, as well as the Internet usage rate according to regions in Thailand (range, 46.2 to 74.6%), which was derived from the National Statistical Office, Ministry of Information and Communication Technology, Thailand, 2020 (24, 30). Additional sensitivity analyses were employed, including (i) a reanalysis of Tobit and ordinal logistic hierarchical regression analysis based on unweighted data and (ii) linear hierarchical regression analysis to reaffirm the main analysis.

The significance level was set at *p* < 0.05 for two-tailed tests. Because we restricted the analysis to participants who completed the intended questionnaires, no imputation was required. All statistical analyses were performed using Stata, version 18.5 (StataCorp LLC, TX, USA).

## Results

### Description of participants and SEP index

Eligible surveys were completed by 1,992 individuals (Supplementary Figure S1). The majority of participants were female (59.8%), with a mean age of 34.8 ± 9.7 years. Table 2 illustrates the sociodemographic, economic, and psychosocial burdens related to the COVID-19 pandemic for all respondents and separately for respondents in each SEP index group. The correlation among the SEP components ranged from 0.32 to 0.61 (Supplementary Figure S2), indicating moderate correlations among the four SEP components (i.e., educational level, personal income, occupational position, and household overcrowding). This suggests that the components share common variance consistent with a general socioeconomic gradient while also capturing distinct aspects of socioeconomic advantages/disadvantages (i.e., they are not interchangeable).

**Table 2:** Participant characteristics according to the SEP index.

Variable	SEP Index Score					*p* value
	Overall (*n* = 1,992)	Very Low: 0–2 Points (*n* = 186)	Low: 3–4 Points (*n* = 614)	Moderate: 5–6 Points (*n* = 998)	High: 7 Points (*n* = 194)	
SEP component^a^						
Educational level						
Low	87 (4.4)	75 (40.3)	12 (2.0)	0 (0.0)	0 (0.0)	<0.001
Medium	306 (15.4)	93 (50.0)	171 (27.8)	42 (4.2)	0 (0.0)	
High	1,599 (80.2)	18 (9.7)	431 (70.2)	956 (95.8)	194 (100.0)	
Personal income, baht/month						
<10,000	177 (8.9)	117 (62.9)	60 (9.8)	0 (0.0)	0 (0.0)	<0.001
10,000–20,000	882 (44.3)	67 (36.0)	524 (85.3)	291 (29.2)	0 (0.0)	
>20,000	933 (46.8)	2 (1.1)	30 (4.9)	707 (70.8)	194 (100.0)	
Occupational position						
Routine/manual	889 (44.6)	185 (99.5)	575 (93.6)	129 (12.9)	0 (0.0)	<0.001
Intermediate	907 (45.6)	1 (0.5)	39 (6.4)	867 (86.9)	0 (0.0)	
Higher/professional	196 (9.8)	0 (0.0)	0 (0.0)	2 (0.2)	194 (100.0)	
Household overcrowding						
Yes	188 (9.4)	176 (94.6)	12 (2.0)	0 (0.0)	0 (0.0)	<0.001
No	1,804 (90.6)	10 (5.4)	602 (98.0)	998 (100.0)	194 (100.0)	
Sociodemographic						
Age in years, mean (SD); median (min – max)	34.8 (9.7); 33 (18–60)	35.6 (10.5); 35 (18–60)	33.0 (8.9); 31 (18–60)	34.3 (9.2); 32 (20–60)	42.2 (10.7); 41 (22–60)	0.001
18–30	812 (40.8)	67 (36.0)	295 (48.0)	424 (42.5)	26 (13.4)	<0.001
31–40	678 (34.0)	56 (30.1)	209 (34.0)	345 (34.6)	68 (35.0)	
41–50	317 (15.9)	45 (24.2)	74 (12.1)	154 (15.4)	44 (22.7)	
51–60	185 (9.3)	18 (9.7)	36 (5.9)	75 (7.5)	56 (28.9)	
Sexual identity						
Male	743 (37.3)	59 (31.7)	204 (33.2)	386 (38.7)	94 (48.5)	0.001
Female	1,191 (59.8)	120 (64.5)	385 (62.7)	588 (58.9)	98 (50.5)	
Others	58 (2.9)	7 (3.8)	25 (4.1)	24 (2.4)	2 (1.0)	
Marital status						
Single	1,301 (65.3)	93 (50.0)	436 (71.0)	682 (68.3)	90 (46.4)	<0.001
Married/domestic partnership	610 (30.6)	81 (43.6)	153 (24.9)	288 (28.9)	88 (45.4)	
Divorced/widowed/separated	81 (4.1)	12 (6.4)	25 (4.1)	28 (2.8)	16 (8.2)	
Religion						
No religion	138 (6.9)	9 (4.8)	31 (5.1)	87 (8.7)	11 (5.7)	0.062
Buddhist	1,779 (89.3)	167 (89.8)	557 (90.7)	878 (88.0)	177 (91.2)	
Christian/Muslim/others	75 (3.8)	10 (5.4)	26 (4.2)	33 (3.3)	6 (3.1)	
Region of residence						
Capital and vicinity	774 (38.9)	27 (14.5)	157 (25.6)	480 (48.1)	110 (56.7)	<0.001
Provincial	1,218 (61.1)	159 (85.5)	457 (74.4)	518 (51.9)	84 (43.3)	
Living status						
Alone	349 (17.5)	12 (6.5)	85 (13.8)	219 (21.9)	33 (17.0)	<0.001
With family	1,492 (74.9)	165 (88.7)	463 (75.4)	706 (70.7)	158 (81.4)	
With others	151 (7.6)	9 (4.8)	66 (10.8)	73 (7.4)	3 (1.6)	
Reimbursement scheme						
Government/state enterprises	376 (18.9)	5 (2.7)	49 (8.0)	270 (27.1)	52 (26.8)	<0.001
Universal coverage scheme	355 (17.8)	93 (50.0)	178 (29.0)	75 (7.5)	9 (4.6)	
Social security scheme	949 (47.6)	63 (33.9)	311 (50.6)	505 (50.6)	70 (36.1)	
Self-payment/others	312 (15.7)	25 (13.4)	76 (12.4)	148 (14.8)	63 (32.5)	
History of mental illness						
No	1,851 (92.9)	170 (91.4)	574 (93.5)	926 (92.8)	181 (93.3)	0.780
Yes	141 (7.1)	16 (8.6)	40 (6.5)	72 (7.2)	13 (6.7)	
History of chronic NCD^b^						
No	1,612 (80.9)	129 (69.4)	516 (84.0)	818 (82.0)	149 (76.8)	<0.001
Yes	380 (19.1)	57 (30.6)	98 (16.0)	180 (18.0)	45 (23.2)	
Economic burden and psychosocial aspects of the COVID-19 pandemic						
Job loss						
No	1,932 (97.0)	167 (89.8)	588 (95.8)	986 (98.8)	191 (98.4)	<0.001
Yes	60 (3.0)	19 (10.2)	26 (4.2)	12 (1.2)	3 (1.6)	
Income loss						
No	1,096 (55.0)	54 (29.0)	260 (42.4)	655 (65.6)	127 (65.5)	<0.001
Monthly income loss of <50%	354 (17.8)	19 (10.2)	100 (16.2)	195 (19.6)	40 (20.6)	
Monthly income loss of ≥50%	542 (27.2)	113 (60.8)	254 (41.4)	148 (14.8)	27 (13.9)	
Debt						
No	562 (28.2)	54 (29.0)	176 (28.7)	280 (28.1)	52 (26.8)	<0.001
Formal debt	1,315 (66.0)	102 (54.8)	376 (61.2)	698 (69.9)	139 (71.6)	
Informal debt	115 (5.8)	30 (16.2)	62 (10.1)	20 (2.0)	3 (1.6)	
Psychosocial issues, mean (SD); median (min – max)						
Perceived stress—PSS 10-item	16.5 (6.5); 17 (0–40)	17.6 (6.4); 17.5 (3–35)	17.5 (6.1); 18 (1–38)	16.3 (6.3); 17 (0–36)	13.2 (7.5); 12 (0–40)	0.003
Resilient coping—BRCS 4-item	13.9 (3.2); 14 (4–20)	12.9 (3.6); 12.5 (4–20)	13.3 (3.3); 14 (4–20)	14.2 (3.0); 15 (4–20)	15.3 (2.8); 16 (4–20)	<0.001
Wellbeing—WHO wellbeing index 5-item	54.7 (21.6); 60 (0–100)	50.0 (23.9); 52 (0–100)	53.3 (21.6); 56 (0–100)	55.0 (20.8); 56 (0–100)	62.2 (20.8); 68 (0–100)	0.085

Data are expressed as the number (percentage) of participants, unless otherwise indicated.

a Details are presented in Table 1.

b To include hypertension, dyslipidemia, diabetes mellitus, stroke and heart disease, chronic kidney disease, cancer, and chronic lung disease.

BRCS, Brief Resilient Coping Scale; COVID-19, coronavirus disease-2019; NCD, non-communicable diseases; PSS, Perceived Stress Scale; SD, standard deviation; SEP, socioeconomic position; WHO, World Health Organization.

Regarding the SEP component (Table 2), the majority of the samples comprised individuals with a high education level (80.3%), personal income >20,000 baht/month (46.8%), intermediate occupational position (45.5%), and household overcrowding (9.4%), with SEP index values of 4.7 ± 1.6 (range, 0–7). Based on the national population and Internet use rate weighted, the sex- and age-adjusted prevalence estimate was 9.8% (95% CI, 8.0–12.0) for very low, 31.6% (95% CI, 28.8–34.6) for low, 48.0% (95% CI, 45.0–51.0) for moderate, and 10.6% (95% CI, 8.7–12.6) for high SEP index groups. Statistical differences in participant characteristics were observed, particularly between the very low and high SEP index groups.

No multicollinearity was observed in the final analysis model (Supplementary Table S1). Moreover, the parallel line assumption for all ordinal logistic regression models was supported by the Brant test (all global tests with *p* > 0.05), indicating no substantial violations of the proportional odds assumption for the SEP index.

### SEP index and perceived stress

The SEP index was associated with a higher PSS score, particularly in the very low SEP index group (Supplementary Figure S3). In the Tobit regression analysis, the significant association between the SEP index category and PSS score was consistent in both the unadjusted and hierarchically adjusted analyses (Models 1 and 2; Table 3 and Supplementary Table S2). Based on the full model adjustment (Model 2), those in the moderate (SEP index 5–6 points), low (SEP index 3–4 points), and very low (SEP index 0–2 points) groups had significantly higher PSS scores than those in the high SEP index group (SEP index = 7 points). In comparison (using a high SEP index as the reference group), the adjusted *β* coefficients were 1.96 (95% CI, 0.71 to 3.21) for moderate, 2.17 (95% CI, 0.77 to 3.56) for low, and 2.94 (95% CI, 1.07 to 4.81) for the very low group.

**Table 3:** Multivariable Tobit regression analysis of SEP index and psychosocial issues-related COVID-19 pandemic (*n* = 1,992).^*^

SEP index category	Perceived stress—PSS score				Resilient coping—BRCS score				Wellbeing—WHO wellbeing score			
	Model 1^a^		Model 2^b^		Model 1^a^		Model 2^b^		Model 1^a^		Model 2^b^	
	*β* coefficient (95% CI)	*p* value	*β* coefficient (95% CI)	*p* value	*β* coefficient (95% CI)	*p* value	*β* coefficient (95% CI)	*p* value	*β* coefficient (95% CI)	*p* value	*β* coefficient (95% CI)	*p* value
High (7 points)	Reference		Reference		Reference		Reference		Reference		Reference	
Moderate (5–6 points)	1.97 (0.68 to 3.26)	0.003	1.96 (0.71 to 3.21)	0.002	−0.74 (−1.37 to −0.10)	0.022	−0.74 (−1.38 to −0.10)	0.024	−5.41 (−10.27 to −0.54)	0.029	−5.40 (−10.17 to −0.63)	0.027
Low (3–4 points)	2.24 (0.81 to 3.66)	0.002	2.17 (0.77 to 3.56)	0.002	−1.48 (−2.21 to −0.75)	<0.001	−1.64 (−2.39 to −0.90)	<0.001	−4.96 (−10.18 to 0.26)	0.062	−5.44 (−10.59 to −0.28)	0.039
Very low (0–2 points)	2.98 (1.12 to 4.84)	0.002	2.94 (1.07 to 4.81)	0.002	−1.12 (−2.15 to −0.08)	0.034	−1.42 (−2.41 to −0.42)	0.005	−10.38 (−17.25 to −3.50)	0.003	−11.52 (−18.26 to −4.77)	0.001

The *β* coefficients corresponding to 95% CIs are weighted according to the national population and Internet use rate in Thailand in 2020.

^*^For perceived stress, *β* coefficients >0 indicate that the lower SEP index groups had a higher risk of stress. Meanwhile, for psychological resilience and wellbeing, *β* coefficients <0 indicate that the lower SEP groups had a higher risk of low resilience to cope and poor wellbeing, respectively.

a Model 1 adjusted for age, sexual identity, marital status, religion, region of residence, living status, reimbursement scheme, history of mental illness, and non-communicable diseases.

b Model 2 adjusted for Model 1 plus job loss, income loss, and debt.

BRCS, Brief Resilient Coping Scale; CI, confidence interval; COVID-19, coronavirus disease-2019; PSS, Perceived Stress Scale; SEP, socioeconomic position; WHO, World Health Organization.

**Figure 1:**
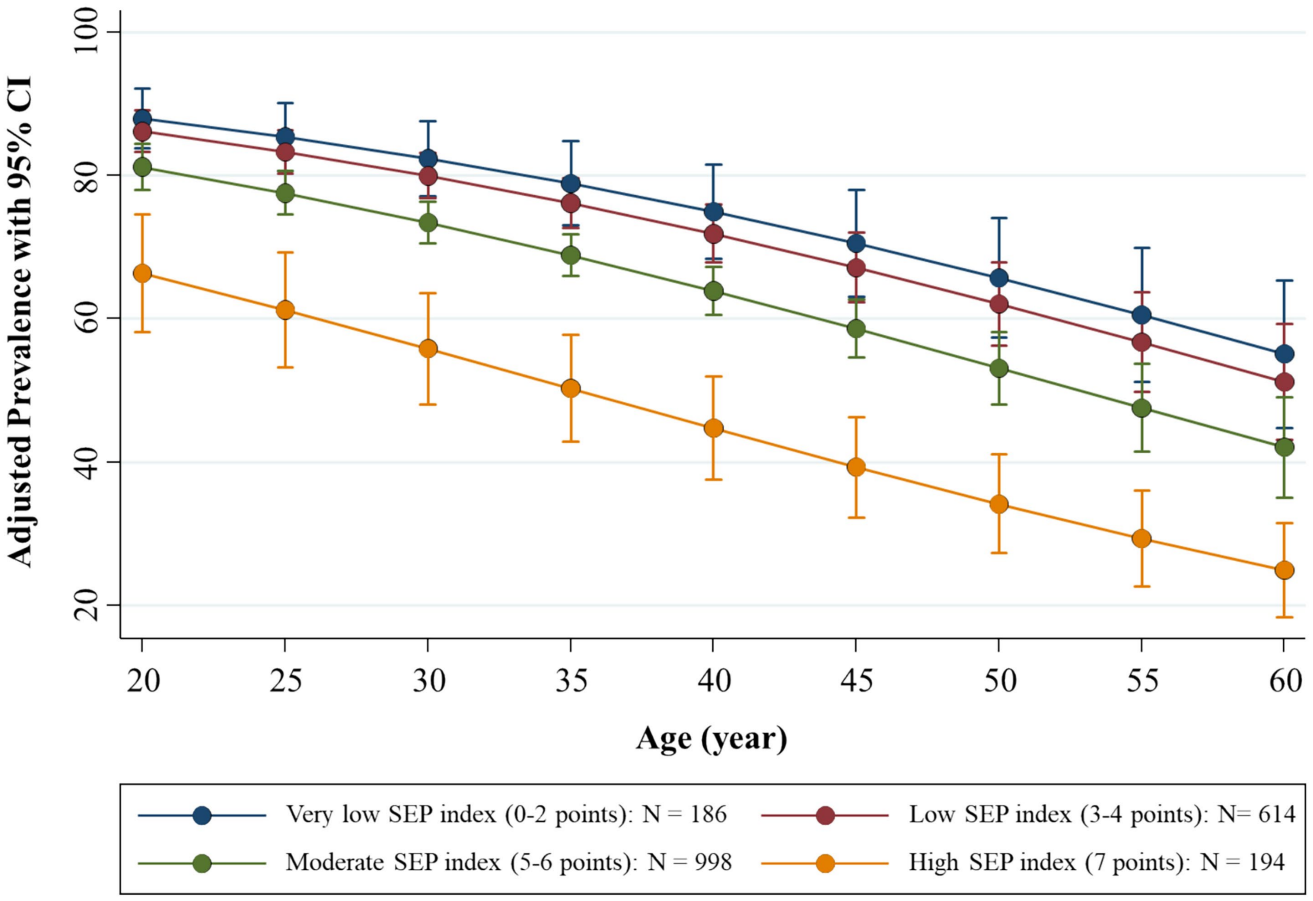
Age and sexual identity adjusted prevalence rates of moderate/high perceived stress (PSS 10-item ≥14) according to the SEP index of participants. The adjusted prevalence corresponding to 95% CIs is weighted according to the national population and internet use rate in Thailand in 2020. CI, confidence interval; PSS, perceived stress scale; SEP, socioeconomic position.

In the ordinal logistic regression analysis, the SEP index category was also significantly associated with a higher risk of perceived stress across the SEP, particularly in the very low SEP index group. Based on the full model, the adjusted common ORs for being worse in terms of perceived stress (three-level severity strata) were 1.90 (95% CI, 1.18–3.04) for moderate, 1.97 (95% CI, 1.17–3.34) for low, and 2.78 (95% CI, 1.34–5.79) for very low compared to those in the high SEP index group (Table 4 and Supplementary Table S2). More importantly, participants in the high SEP index group had a substantially lower prevalence of moderate/high perceived stress (PSS 10-item ≥14) across all age groups than participants in the other groups (Figure 1).

**Table 4:** Multivariable ordinal logistic regression analysis of SEP index and psychosocial issues-related COVID-19 pandemic (*n* = 1,992).^*^

SEP index category^c^	Perceived stress [reference category, none/minimal perceived stress (PSS ≤ 13)]				Resilient coping [reference category, low resilient copers (BRCS ≤13)]				Wellbeing [reference category, very poor wellbeing (WHO wellbeing index ≤2)]			
	Model 1^a^		Model 2^b^		Model 1^a^		Model 2^b^		Model 1^a^		Model 2^b^	
	Common OR (95% CI)	*p* value	Common OR (95% CI)	*p* value	Common OR (95% CI)	*p* value	Common OR (95% CI)	*p* value	Common OR (95% CI)	*p* value	Common OR (95% CI)	*p* value
High (7 points)	Reference		Reference		Reference		Reference		Reference		Reference	
Moderate (5–6 points)	1.90 (1.18–3.04)	0.008	1.90 (1.20–3.02)	0.003	0.74 (0.48–1.13)	0.165	0.74 (0.48–1.14)	0.171	0.55 (0.33–0.93)	0.027	0.56 (0.33–0.95)	0.030
Low (3–4 points)	1.97 (1.17–3.34)	0.011	2.03 (1.20–3.44)	0.009	0.48 (0.30–0.78)	0.002	0.44 (0.27–0.71)	0.001	0.59 (0.34–1.03)	0.062	0.58 (0.33–1.01)	0.054
Very low (0–2 points)	2.78 (1.34–5.79)	0.006	3.02 (1.45–6.33)	0.003	0.51 (0.26–1.01)	0.052	0.44 (0.23–0.86)	0.016	0.33 (0.17–0.65)	0.001	0.30 (0.16–0.58)	<0.001

The common ORs corresponding to 95% CIs are weighted according to the national population and Internet use rate in Thailand in 2020.

^*^For perceived stress, ORs > 1 indicate that the lower SEP index groups had a higher risk of stress. Meanwhile, for psychological resilience and wellbeing, ORs < 1 indicated that the lower SEP groups had a higher risk of poor coping resilience and poor wellbeing, respectively.

a Model 1 adjusted for age, sexual identity, marital status, religion, region of residence, living status, reimbursement scheme, history of mental illness, and non-communicable diseases.

b Model 2 adjusted for Model 1 plus job loss, income loss, and debt.

c Based on three-level categories: perceived stress (none/minimal, moderate, high); psychological resilience (low, medium, high); and wellbeing (very poor, poor, fair/high).

BRCS, Brief Resilient Coping Scale; CI, confidence interval; COVID-19, coronavirus disease-2019; OR, odds ratio; PSS, Perceived Stress Scale; SEP, socioeconomic position; WHO, World Health Organization.

### SEP index and resilient coping

Supplementary Figure S3 shows a proportionally positive relationship between the SEP index scores and BRCS scores. In Tobit regression analysis (Table 3), the adjusted *β* coefficients (full model) were −0.74 (95% CI, −1.38 to −0.10) for moderate, −1.64 (95% CI, −2.39 to −0.90) for low, and −1.42 (95% CI, −2.41 to −0.42) for the very low group compared to those in the high SEP index group (unadjusted model is provided in Supplementary Table S2).

**Figure 2:**
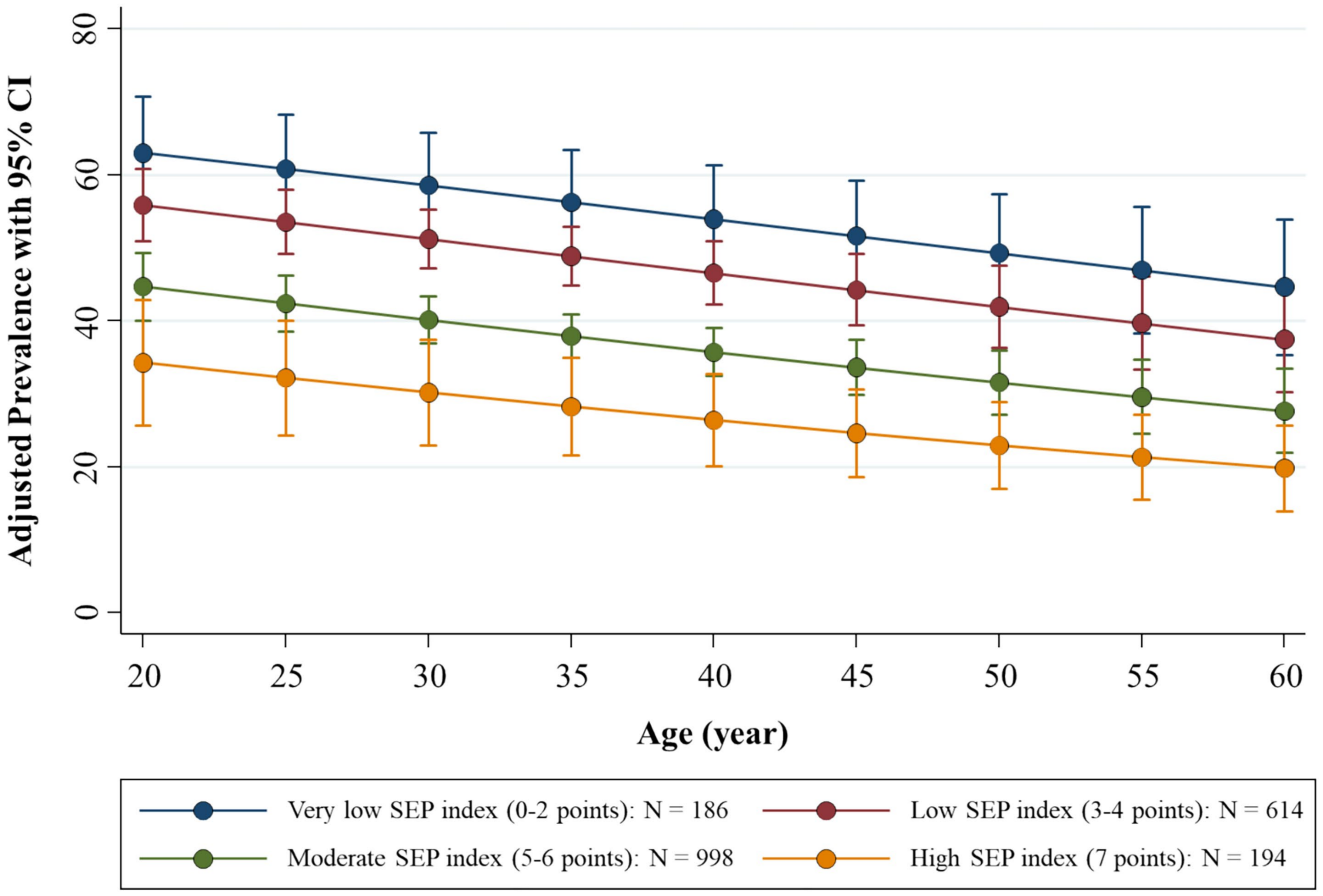
Age and sexual identity adjusted prevalence rates of low resilience copers (BRCS 4-item ≤13) according to the SEP index of participants. The adjusted prevalence corresponding to 95% CIs is weighted according to the national population and the internet use rate in Thailand in 2020. BRCS, Brief Resilient Coping Scale; CI, confidence interval; SEP, socioeconomic position.

In comparison with the high SEP index group, the ordinal logistic regression based on the full model found significantly lower ORs for being high-resilient copers (three-level strata) only in the low and very low SEP index groups: the common ORs were 0.44 (95% CI, 0.27–0.71) and 0.44 (95% CI, 0.23–0.86), respectively (Table 4; unadjusted model is provided in Supplementary Table S2). Furthermore, across age groups, participants in the high SEP index group showed a trend toward a lower prevalence of low-resilient copers (BRCS 4-item ≤13) than those in the other groups (Figure 2).

### SEP index and wellbeing

Similarly, a proportionally positive relationship between the SEP index scores and WHO wellbeing scores is similar to the pattern observed for psychological resilience (Supplementary Figure S3). Compared with the high SEP index group, the adjusted *β* coefficients based on the full model of Tobit regression analysis were −5.40 (95% CI, −10.17 to −0.63) for moderate, −5.44 (95% CI, −10.59 to −0.28) for low, and −11.52 (95% CI, −18.26 to −4.77) for the very low SEP index group (Table 3; the unadjusted model is provided in Supplementary Table S2).

**Figure 3:**
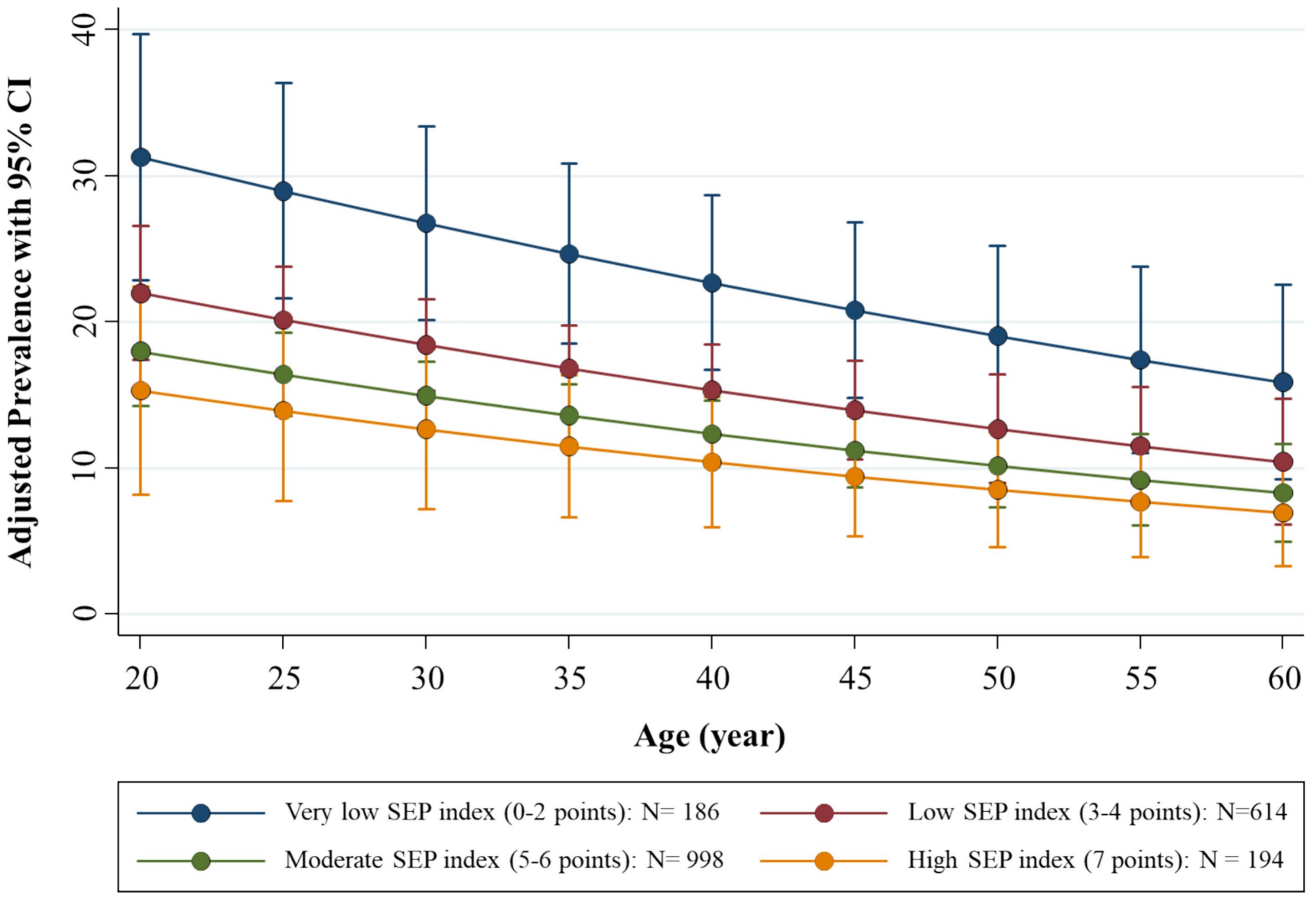
Age and sexual identity adjusted prevalence rates of very poor wellbeing (WHO wellbeing index ≤28) according to the SEP index of participants. The adjusted prevalence corresponding to 95% CIs is weighted according to the national population and the internet use rate in Thailand in 2020. CI, confidence interval; SEP, socioeconomic position; WHO, World Health Organization.

In the ordinal logistic regression (full model, Table 4; the unadjusted model is provided in Supplementary Table S2), significant associations with fair/high wellbeing index (three-level strata) were observed for the moderate and very low SEP index groups. The adjusted common ORs were 0.56 (95% CI, 0.33–0.95) and 0.30 (95% CI, 0.16–0.58) for the moderate and very low SEP index groups, respectively, compared with the high SEP index group. Meanwhile, the estimate for the low SEP index group was borderline and did not reach conventional statistical significance (full model, Table 4; adjusted common OR, 0.58; 95% CI, 0.33–1.01; *p* = 0.054). Additionally, across age groups, participants in the high SEP index group appeared to have a lower prevalence of very poor wellbeing (WHO wellbeing index 5-item ≤28) than those in the other groups (Figure 3).

### Sensitivity analysis

After using an unweighted Tobit and ordinal logistic hierarchical regression model, our findings remained identical to those of the main analysis (Supplementary Tables S3, S4). Additional analyses using linear hierarchical regression also confirmed a linear relationship between the SEP index scores and the risk of adverse psychosocial issues (Supplementary Table S5).

## Discussion

### Overview of findings

In the general population of Thailand, this study was the first to propose a simple individual SEP index (a composite of four variables: education level, personal income, occupational position, and housing overcrowding) using a public mental health survey during the early recession brought about by the COVID-19 pandemic. Overall, the majority of participants were classified as moderate (48.0%), followed by low (31.6%) SEP indices, whereas only 9.8 and 10.5% were classified as very low and high SEP indices, respectively. Our findings showed that individuals with low SEP index scores were at significantly higher risk of adverse psychosocial issues. Interestingly, compared with the high SEP index group, those with lower SEP index scores, particularly those in the very low SEP index group, had a significantly higher risk of moderate/high perceived stress, low resilient coping, and very poor wellbeing.

### Comparison with previous studies

During the COVID-19 pandemic, previous studies have generally focused on the relationship between SEP gradients and non-mental health and psychosocial issues (e.g., non-communicable diseases or healthcare utilization), on the non-working-aged population, or on non-validated measurement tools for capturing psychological responses. In the early weeks of lockdown in the United Kingdom, Wright et al. revealed that the SEP gradient was associated with adversities-related finances (i.e., income and job loss) and basic needs (i.e., access to food and medications) (31). These findings were also supported by a report from the Vienna population from April to May 2020, which found that individuals in the lowest SEP category had a higher incidence of adverse health-related and socioeconomic outcomes (incidence rate ratio, 1.33; 95% CI, 1.08–1.64) (32). Moreover, in three waves of the pandemic (March to December 2020) in Spain, Aguilar-Palacio et al. found that socioeconomic factors, as measured by the index of individual and basic healthcare area of residence levels, were associated with an increased risk of COVID-19-confirmed infection (15).

Several studies have examined the association between SEP gradients, using area deprivation indices or family SEP, and adverse psychological outcomes in a particular population of non-working-aged individuals. For example, a study by Myhr et al. using repeated cross-sectional surveys of Norwegian adolescents reported that low family SEP was associated with higher risks of depressive symptoms and loneliness in girls (OR, 1.66; *p* < 0.001 and 1.90; *p* < 0.001) and depressive symptoms in boys (OR, 2.33; *p* < 0.001), compared to those with high family SEP (33). Moreover, the authors indicated that adolescents from high-SEP families also reported higher life satisfaction. However, adjustment for potential confounders was limited, potentially affecting the interpretation of these associations.

Regarding stress-related COVID-19 infection, Hammett et al. illustrated the association between COVID-19 stress and physically intimate partner aggression, particularly among individuals living in lower areas with socioeconomic deprivation (16). Using non-validated measurement tools, a report from the Household Pulse Survey during the COVID-19 pandemic found that United States adults with lower income, lower education, and renters had statistically significantly higher fair or poor health status and experience with serious depressive symptoms compared to individuals with higher SEP (ORs range from 1.06 to 4.98) (34). Correspondingly, an extended survey based on the Household Pulse Survey from September 2022 to February 2023 also reported that inflation stress varied according to sociodemographic characteristics such as sex, race and ethnicity, education, and income levels (35).

Our findings revealed that a lower SEP index was associated with reduced psychological resilience and poorer wellbeing, aligning with international evidence that socioeconomic disadvantage constrains coping resources and undermines subjective wellbeing (7, 8, 13). Although resilience can protect against mental health problems, it is itself shaped by social determinants of health (e.g., education, income, occupation), and the lower WHO wellbeing scores observed among individuals with a low SEP index likely reflect the cumulative burden of economic and social stressors during the COVID-19 pandemic. Following ordinal outcome analysis, the three levels of the WHO wellbeing index were generally associated across the SEP index groups, except for the low SEP group (borderline effect estimate). This may reflect reduced sensitivity after categorizing the WHO wellbeing index into three categories and/or limited power to detect differences between adjacent categories. Analyses using the continuous WHO wellbeing score revealed a more consistent graded pattern.

Unfortunately, the majority of quantitative evidence on SEP gradients in psychosocial outcomes during the COVID-19 pandemic comes from Europe and North America, and pathways may differ in Asian contexts because of distinct labor markets, welfare institutions, and family support systems. Cross-cultural work in East and Southeast Asia highlights the role of unequal resources and access, particularly in shaping digital inclusion and social participation, as well as coping capacity and wellbeing (14). In Thailand, the reimbursement scheme may reduce the insecurity of access to healthcare under universal health coverage. Meanwhile, a large informal sector may heighten economic vulnerability during downturns, and strong household/intergenerational support norms may either buffer or compound stress through shared financial and caregiving responsibilities. These contextual factors may shape both exposure to pandemic stressors and the availability of coping resources, thereby influencing the magnitude and pattern of SEP gradients in resilience and wellbeing.

Collectively, we have expanded and closed this gap with previous SEP gradient studies by focusing on psychosocial issues using well-validated measurement tools in a public population of working-age adults. Our findings highlight the social value of the SEP index in public mental health surveys and practices. The proposed SEP index scores are based on four simple indicators that can quantify and reflect adverse psychosocial issues, particularly among those in the lower SEP index categories.

### Strengths and limitations

This study has strengths, as it reflects the public’s psychosocial responses to stress, psychological resilience, and wellbeing during the early phase of the COVID-19 pandemic and economic downturns in Thailand. The proposed SEP index and its categorization strata are simple, practical, ready-to-use, and can be incorporated into public mental health surveys. Furthermore, our findings were robust and consistent across different statistical analyses (Tobit and ordinal logistic hierarchical regression analyses using both weighted and unweighted national population and the rate of Internet use in Thailand).

Our study has certain limitations. First, the cross-sectional study design and the lack of a comprehensive mental health assessment could limit our ability to establish causality and assess the longitudinal effects of the results. Nevertheless, SEP status is a time-invariant variable that is unlikely to produce profound changes in the findings. We also postulated that the relationship between SEP gradient and psychosocial issues appears to be bidirectional rather than causal.

Second, this analysis was conducted during the first wave of the COVID-19 pandemic (April–May 2020) in Thailand, providing only an early snapshot of the pandemic. Psychosocial impacts may intensify over time as stress accumulates and uncertainty persists. Therefore, the magnitude of associations, particularly for resilience and wellbeing, may differ in later phases when economic disruption and adaptation fatigue are more pronounced. To implement a public mental health survey, repeated measurements in subsequent waves, including temporal and external validation of the proposed SEP index, are needed to assess whether SEP-related disparities have widened over the course of the pandemic.

Third, although we used well-validated instruments and theoretically sound methods to capture psychological responses during the COVID-19 pandemic, the limitations of using self-reported measures should be noted. The SEP index was designed to be simple and feasible for routine public health monitoring. However, the equal-weight additive approach may not accurately reflect the empirically optimal contribution of each SEP dimension. Future studies could compare this approach with data-driven methods (e.g., factor analysis, latent class analysis, or item response theory) to assess whether a latent SEP index improves measurement while accounting for trade-offs in interpretability and cross-study comparability. Furthermore, context-specific calibration (e.g., by residency area [rural, suburbanized, and urbanized communities] or by applying alternative thresholds of the SEP index) and validation against external socioeconomic benchmarks should be explored.

Fourth, although we accounted for several potential confounding factors regarding sociodemographic and economic burdens related to the COVID-19 pandemic, residual unmeasured variables such as health literacy, risk perception, self-care behaviors related to the COVID-19 pandemic, details of housing (e.g., housing tenure), household income, food insecurity, access to transportation, and neighborhood safety should be addressed.

Finally, we found a significant relationship (both linear and ordered) between the SEP index and adverse psychosocial outcomes, especially in the very low SEP index group. A relatively small sample size and less diverse populations in this particular group should be acknowledged. As per the study design, using an online cross-sectional nationwide survey to adhere to the physical distancing strategy, participants with barriers to Internet access and those who had economic instability in relation to the direct and indirect SEP gradient (i.e., inequality in physical infrastructure and social and community context, racial/minority segregation, rurality, and social inclusion) (36) may be underrepresented in this study. Indeed, online recruitment excludes individuals without Internet access who are disproportionately socioeconomically disadvantaged. As a result, the “very low SEP index” group in this sample may reflect more connected Internet access and relatively advantaged individuals rather than a true subset of the very low SEP index in the community. Although weighting national population distributions and Internet use rates may reduce this bias, it cannot fully address non-coverage, and SEP gradients, especially those involving the lowest SEP group, may be underestimated. Further studies should employ mixed-mode surveys, both offline and online, to identify the most disadvantaged groups. In such circumstances, selection and respondent biases should also be considered. These factors likely limit the generalizability of our findings to other countries and populations.

### Implications for public mental health

Given these limitations, we underscore the importance of a simple and practical SEP index to understand psychosocial responses during the COVID-19 pandemic and broader economic downturns. The observed SEP gradients suggest an urgent need for mental health action plans and coping support strategies targeted at people with very low or low SEP. Population-level policies could include the early identification of at-risk groups using the SEP index, strengthening local social capital, and scaling community-based coping skills and resilience-building programs. Public mass digital mental health interventions to reduce financial toxicity during recessions (e.g., facilitated access to financial institutions and utility support, financial counseling, and debt-relief programs) are also warranted.

Addressing SEP-related inequities will require coordinated efforts across healthcare providers, managed care organizations, and community mental health networks to integrate screening and referral into public health programming and, where feasible, value-based care and reimbursement mechanisms. Further research should examine how social determinants of health and SEP gradients shape mental health and wellbeing following public health emergencies and should prioritize mixed-mode and longitudinal designs, including more socioeconomically diverse and multinational samples, to improve generalizability and causal inference. Future work should also evaluate the applicability of the SEP index to other mental health outcomes and non-mental health conditions linked to social disadvantages.

## Conclusion

The proposed SEP index is a simple tool for quantifying individuals at risk for psychosocial issues in public health surveys. During the economic downturn and pandemic, we found that the SEP index was associated with perceived stress, psychological resilience, and wellbeing among working-age adults (18–60 years) in the general population in Thailand. Our findings might motivate public health policymakers and stakeholders to address SEP gradients in public mental health programs. However, the proposed individual SEP index should be externally validated in independent Thai samples and against established SEP measures before it can be adopted for routine public health surveys.

## Data Availability

The raw data supporting the conclusions of this article will be made available by the authors, without undue reservation.
