# Supplementary material for: Individual socioeconomic position among the general population and economic downturns-related perceived stress, psychological resilience, and wellbeing in Thailand
Source: Front Public Health. 2026 Jun 8;14:1855202. doi: 10.3389/fpubh.2026.1855202 (PMC13283863; doi:10.3389/fpubh.2026.1855202)
Supplement: Supplementary file 1 [file Supplementary_file_1.pdf]

Supplementary Online Information

Individual Socioeconomic Position Among the General Population and Economic Downturns-Related Perceived Stress, Psychological Resilience, and Well-Being in Thailand

Ratanaporn Awiphan, Chidchanok Ruengorn, Chabaphai Phosuya, Kiatkriangkrai Koyratkoson, Penkarn Kanjanarat, Kednapa Thavorn, Nahathai Wongpakaran, Tinakon Wongpakaran, Surapon Nochaiwong

Correspondence and requests for materials:
Surapon Nochaiwong, PharmD, Department of Pharmaceutical Care, Faculty of Pharmacy, Chiang Mai University, Chiang Mai 50200, Thailand, Phone: +6653944342, Fax: 6653944390, Email: surapon.nochaiwong@gmail.com
X (formerly Twitter): @PESRC_CMU

Supplementary Online Content

**Table S1**	Collinearity Diagnostics	S3
**Table S2**	Unadjusted Analysis of Association Between SEP Index and Psychosocial Issues-Related COVID-19 Pandemic	S4
**Table S3**	Unweighted Multivariable Tobit Regression Analysis of SEP Index and Psychosocial Issues-Related COVID-19 Pandemic	S5
**Table S4**	Unweighted Multivariable Ordinal Logistic Regression Analysis of SEP Index and Psychosocial Issues-Related COVID-19 Pandemic	S6
**Table S5**	Multivariable Linear Regression Analysis of SEP Index and Psychosocial Issues-Related COVID-19 Pandemic	S7
**Figure S1**	Flow Diagram for Study Participants	S8
**Figure S2**	Correlation Matrix of the SEP Components	S9
**Figure S3**	Differences in Psychological Issue Scores According to the SEP Index	S10
**Appendix S1**	Description of the Questionnaires	S11

Table S1 Collinearity Diagnostics

Variables	VIF*	Tolerance§
Age	1.72	0.58
Sexual identity	1.12	0.89
Marital status	1.58	0.63
Religion	1.05	0.96
Region of residence	1.13	0.88
Living status	1.12	0.90
Reimbursement scheme	1.12	0.89
History of mental illness	1.03	0.98
History of chronic non-communicable diseases	1.17	0.86
Job loss	1.08	0.92
Income loss	1.14	0.88
Debt	1.10	0.91
Mean VIF	1.19	

*VIF <2.5 indicates lack of collinearity.
§Tolerance is the reciprocal of VIF, with a value of 0.40 or less is cause for concern.
Abbreviation: VIF, variance inflation factor.

Table S2 Unadjusted Analysis of Association Between SEP Index and Psychosocial Issues-Related COVID-19 Pandemic*

**Tobit Regression Analysis**	**Perceived stress—PSS Score**		**Resilient coping—BRCS score**		**Well-being—WHO well-being score**	
	**β Coefficient (95% CI)**	***P* Value**	**β Coefficient (95% CI)**	***P* Value**	**β Coefficient (95% CI)**	***P* Value**
SEP index category						
7 points	Reference		Reference		Reference	
5 – 6 points	3.15 (2.15 to 4.14)	<0.001	-1.07 (-1.58 to -0.56)	<0.001	-7.09 (-10.43 to -3.74)	<0.001
3 – 4 points	4.42 (3.37 to 5.46)	<0.001	-2.04 (-2.57 to -1.51)	<0.001	-8.94 (-12.46 to -5.43)	<0.001
0 – 2 points	4.52 (3.22 to 5.81)	<0.001	-2.40 (-3.06 to -1.74)	<0.001	-12.26 (-16.64 to -7.88)	<0.001
**Ordinal Logistic Regression Analysis§**	**Perceived stress (reference category, no/minimal perceived stress [PSS ≤13])**		**Resilient coping (reference category, low resilient copers [BRCS ≤13])**		**Well-being (reference category, very poor well-being [WHO well-being index ≤28])**	
	**Common OR (95% CI)**	***P* Value**	**Common OR (95% CI)**	***P* Value**	**Common OR (95% CI)**	***P* Value**
SEP index category						
7 points	Reference		Reference		Reference	
5 – 6 points	2.82 (2.06 – 3.86)	<0.001	0.58 (0.44 – 0.78)	<0.001	0.47 (0.33 – 0.67)	<0.001
3 – 4 points	4.06 (2.91 – 5.67)	<0.001	0.38 (0.28 – 0.51)	<0.001	0.46 (0.32 – 0.66)	<0.001
0 – 2 points	4.59 (3.00 – 7.02)	<0.001	0.31 (0.21 – 0.45)	<0.001	0.32 (0.21 – 0.50)	<0.001

Note: The β coefficients and common ORs corresponding to 95% CIs are weighted according to the national population and the Internet use rate in Thailand, 2020.

*For perceived stress, β coefficients >0 or ORs >1 indicate that the lower SEP index groups had more risk of stress. Meanwhile, for psychological resilience and well-being, β coefficients <0 or ORs <1 indicate that the lower SEP groups had a higher risk of low resilience to cope and poor well-being, respectively.

§Based on three-level categories: perceived stress (no/minimal, moderate, high); psychological resilience (low, medium, high); and well-being (very poor, poor, fair/high).

Abbreviations: BRCS, Brief Resilient Coping Scale; CI, confidence interval; COVID-19, coronavirus disease-2019; OR, odds ratio; PSS, Perceived Stress Scale; SEP, socioeconomic position; WHO, World Health Organization.

Table S3 Unweighted Multivariable Tobit Regression Analysis of SEP Index and Psychosocial Issues-Related COVID-19 Pandemic (n=1,992)*

SEP index category	Perceived stress—PSS score				Resilient coping—BRCS score				Well-being—WHO well-being score			
	Model 1†		Model 2‡		Model 1†		Model 2‡		Model 1†		Model 2‡	
	β Coefficient (95% CI)	*P* Value	β Coefficient (95% CI)	*P* Value	β Coefficient (95% CI)	*P* Value	β Coefficient (95% CI)	*P* Value	β Coefficient (95% CI)	*P* Value	β Coefficient (95% CI)	*P* Value
High (7 points)	Reference		Reference		Reference		Reference		Reference		Reference	
Moderate (5–6 points)	1.74 (0.75 to 2.73)	0.001	1.71 (0.73 to 2.70)	0.001	-0.63 (-1.15 to -0.11)	0.018	-0.64 (-1.16 to -0.13)	0.015	-3.73 (-7.09 to -0.38)	0.029	-3.78 (-7.13 to -0.43)	0.027
Low (3–4 points)	2.61 (1.52 to 3.69)	<0.001	2.42 (1.33 to 3.51)	<0.001	-1.36 (-1.94 to -0.80	<0.00 1	-1.50 (-2.08 to -0.93)	<0.001	-4.34 (-8.01 to -0.66)	0.021	-4.63 (-8.34 to -0.92)	0.014
Very low (0–2 points	2.67 (1.33 to 4.01)	<0.001	2.44 (1.08 to 3.80)	<0.001	-1.62 (-2.32 to -0.91)	<0.00 1	-1.84 (-2.55 to -1.13)	<0.001	-7.33 (-11.90 to -2.76)	0.002	-7.78 (-12.42 to -3.14)	0.001

*For perceived stress, β coefficients >0 indicate that the lower SEP index groups had more risk of stress. Meanwhile, for psychological resilience and well-being, β coefficients <0 indicate that the lower SEP groups had a higher risk of low resilience to cope and poor well-being, respectively.

†Model 1 adjusted for age, sex, marital status, religion, region of residence, living status, reimbursement scheme, history of mental illness and non-communicable diseases.

‡Model 2 adjusted for model 1 plus job loss, income loss, and debt.

Abbreviations: BRCS, Brief Resilient Coping Scale; CI, confidence interval; COVID-19, coronavirus disease-2019; PSS, Perceived Stress Scale; SEP, socioeconomic position; WHO, World Health Organization.

Table S4 Unweighted Multivariable Ordinal Logistic Regression Analysis of SEP Index and Psychosocial Issues-Related COVID-19 Pandemic (n=1,992)*

SEP index category§	Perceived stress (reference category, no/minimal perceived stress [PSS ≤13])				Resilient coping (reference category, low resilient copers [BRCS ≤13])				Well-being (reference category, very poor well-being [WHO well-being index ≤28])			
	**Model 1†**		**Model 2‡**		**Model 1†**		**Model 2‡**		**Model 1†**		**Model 2‡**	
	**Common OR (95% CI)**	***P* Value**	**Common OR (95% CI)**	***P* Value**	**Common OR (95% CI)**	***P* Value**	**Common OR (95% CI)**	***P* Value**	**Common OR (95% CI)**	***P* Value**	**Common OR (95% CI)**	***P* Value**
High (7 points)	Reference		Reference		Reference		Reference		Reference		Reference	
Moderate (5–6 points)	1.91 (1.36 – 2.67)	<0.001	1.92 (1.37 – 2.70)	<0.001	0.74 (0.55 – 1.00)	0.052	0.73 (0.54 – 0.99)	0.044	0.62 (0.42 – 0.90)	0.013	0.62 (0.42 – 0.90)	0.013
Low (3–4 points)	2.47 (1.70 – 3.58)	<0.001	2.49 (1.71 – 3.63)	<0.001	0.53 (0.38 – 0.74)	<0.001	0.49 (0.35 – 0.69)	<0.001	0.64 (0.43 – 0.97)	0.033	0.63 (0.42 – 0.95)	0.026
Very low (0–2 points	2.82 (1.77 – 4.50)	<0.001	2.90 (1.80 – 4.68)	<0.001	0.46 (0.30 – 0.70)	<0.001	0.41 (0.27 – 0.63)	<0.001	0.47 (0.29 – 0.75)	0.002	0.45 (0.28 – 0.73)	0.001

Note: The common ORs corresponding to 95% CIs are weighted according to the national population and the Internet use rate in Thailand, 2020.

*For perceived stress, ORs >1 indicate that the lower SEP index groups had more risk of stress. Meanwhile, for psychological resilience and well-being, ORs <1 indicate that the lower SEP groups had a higher risk of low resilience to cope and poor well-being, respectively.

†Model 1 adjusted for age, sex, marital status, religion, region of residence, living status, reimbursement scheme, history of mental illness and non-communicable diseases.

‡Model 2 adjusted for model 1 plus job loss, income loss, and debt.

§Based on three-level categories: perceived stress (no/minimal, moderate, high); psychological resilience (low, medium, high); and well-being (very poor, poor, fair/high).

Abbreviations: BRCS, Brief Resilient Coping Scale; CI, confidence interval; COVID-19, coronavirus disease-2019; OR, odds ratio; PSS, Perceived Stress Scale; SEP, socioeconomic position; WHO, World Health Organization.

Table S5 Multivariable Linear Regression Analysis of SEP Index and Psychosocial Issues-Related COVID-19 Pandemic (n=1,992)*

**SEP index**	**Perceived stress—PSS Score**				**Resilient coping—BRCS score**				**Well-being—WHO well-being score**			
	**Model 1†**		**Model 2‡**		**Model 1†**		**Model 2‡**		**Model 1†**		**Model 2‡**	
	**β Coefficient (95% CI)**	***P* Value**	**β Coefficient (95% CI)**	***P* Value**	**β Coefficient (95% CI)**	***P* Value**	**β Coefficient (95% CI)**	***P* Value**	**β Coefficient (95% CI)**	***P* Value**	**β Coefficient (95% CI)**	***P* Value**
SEP index category												
High (7 points)	Reference		Reference		Reference		Reference		Reference		Reference	
Moderate (5–6 points)	1.67 (0.69 to 2.66)	0.001	1.65 (0.66 2.63)	0.001	-0.61 (-1.11 to -0.11)	0.016	-0.63 (-1.12 to -0.13)	0.013	-3.88 (-7.17 to -0.58)	0.021	-3.93 (-7.22 to -0.63)	0.019
Low (3–4 points)	2.54 (1.46 to 3.62)	<0.001	2.36 (1.27 to 3.45)	<0.001	-1.33 (-1.88 to -0.79)	<0.001	-1.47 (-2.02 to -0.92)	<0.001	-4.38 (-7.99 to -0.77)	0.017	-4.67 (-8.32 to -1.03)	0.012
Very low (0–2 points	2.61 (1.27 to 3.95)	<0.001	2.38 (1.02 to 3.74)	0.001	-1.59 (-2.26 to -0.91)	<0.001	-1.81 (-2.50 to -1.12)	<0.001	-7.36 (-11.84 to -2.88)	0.001	-7.81 (-12.37 to -3.26)	0.001
SEP index continuous (range, 0-7)	-0.45 (-0.65 to -0.26)	<0.001	-0.41 (-0.61 to -0.21)	<0.001	0.31 (0.22 to 0.41)	<0.001	0.36 (0.26 to 0.46)	<0.001	1.15 (0.49 to 1.80)	0.001	1.25 (0.57 to 1.93)	<0.001

Note: The common ORs corresponding to 95% CIs are weighted according to the national population and the Internet use rate in Thailand, 2020.
*For perceived stress, β coefficients >0 indicate that the lower SEP index groups had more risk of stress. Meanwhile, for psychological resilience and well-being, β coefficients <0 indicate that the lower SEP groups had a higher risk of low resilience to cope and poor well-being, respectively.
†Model 1 adjusted for age, sex, marital status, religion, region of residence, living status, reimbursement scheme, history of mental illness and non-communicable diseases.
‡Model 2 adjusted for model 1 plus job loss, income loss, and debt.
Abbreviations: BRCS, Brief Resilient Coping Scale; CI, confidence interval; COVID-19, coronavirus disease-2019; PSS, Perceived Stress Scale; SEP, socioeconomic position; WHO, World Health Organization.

Figure S1 Flow Diagram for Study Participants

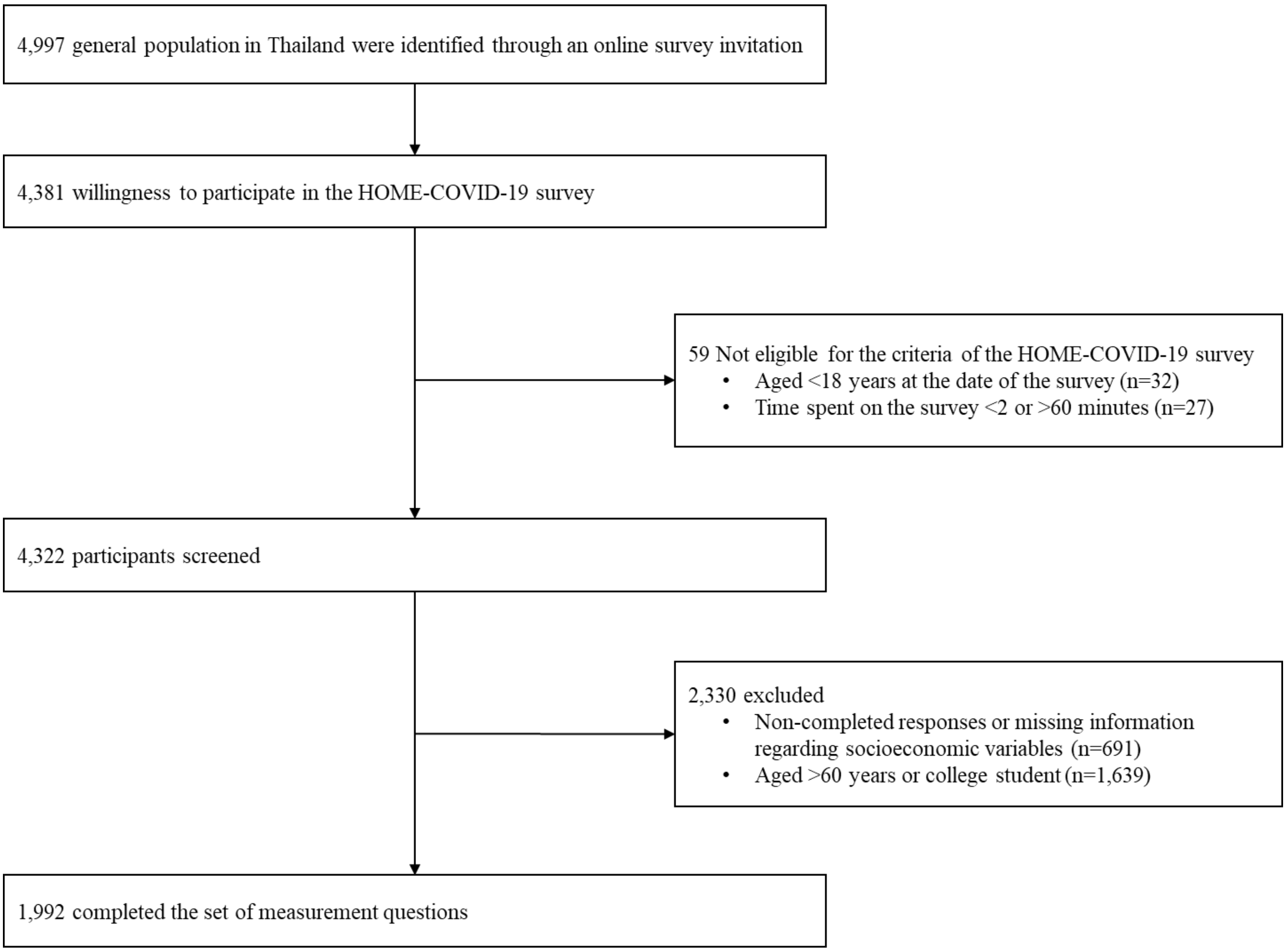

Abbreviation: HOME-COVID-19, the Health Outcomes and Mental Health Care Evaluation Survey Research Group-Coronavirus Disease 2019.

Figure S2 Correlation Matrix of the SEP Components

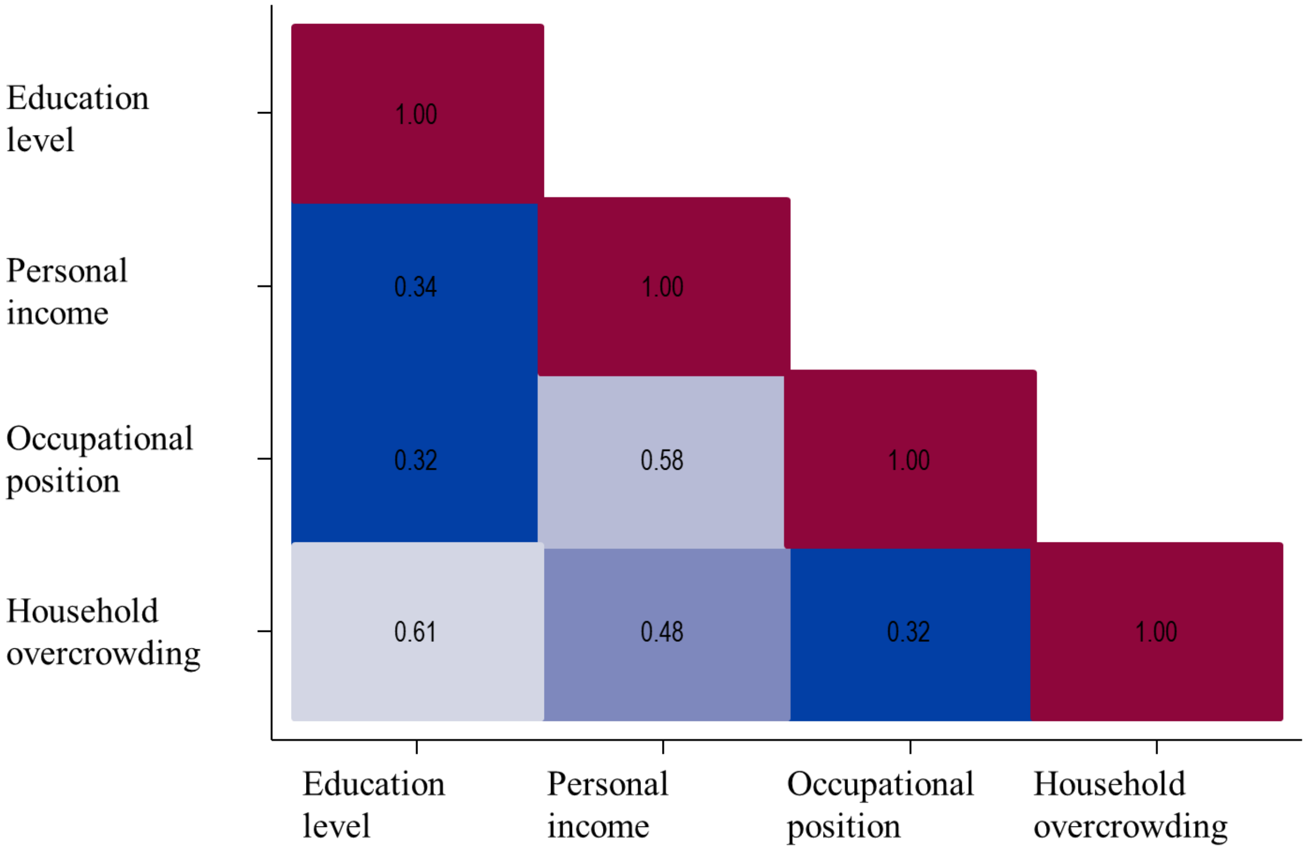

Notes: color indicates the strength of the correlation.
Abbreviation: SEP socioeconomic position.

Figure S3 Differences in Psychological Issue Scores According to the SEP Index

(A) Perceived stress—PSS Score

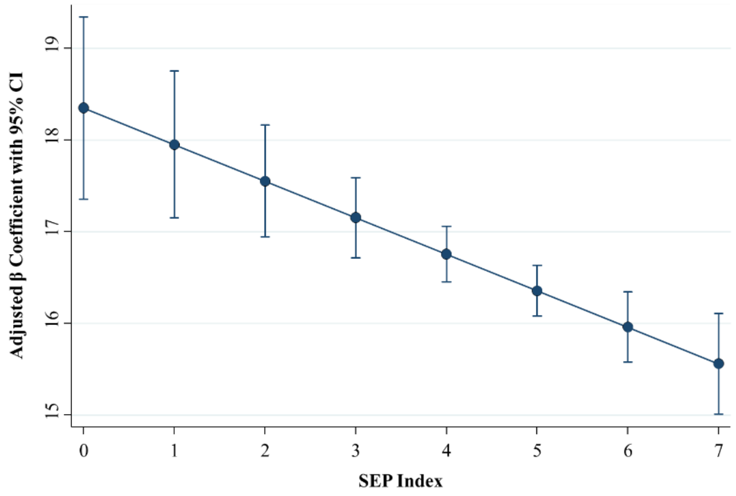

(B) Resilient coping—BRCS score

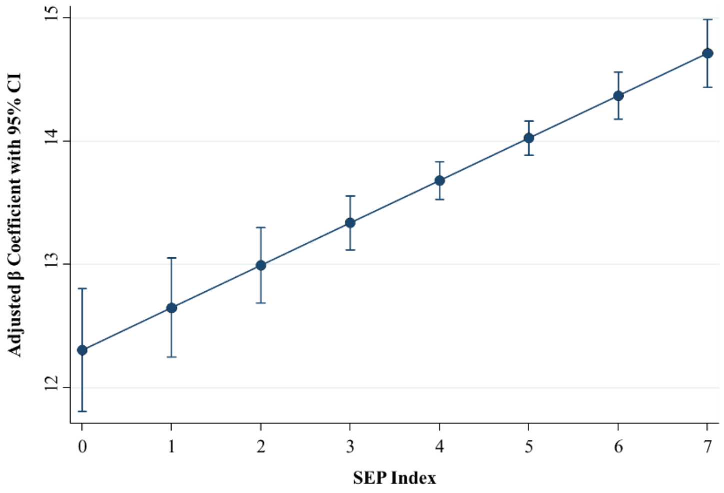

(C) Well-being—WHO well-being score

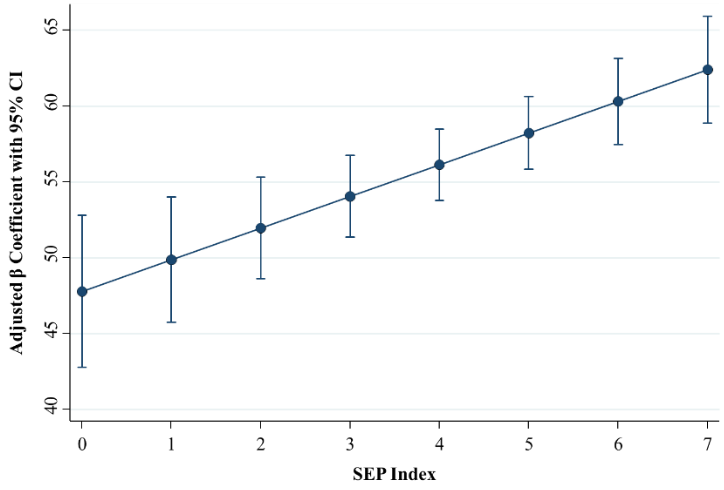

Abbreviations: BRCS, Brief Resilient Coping Scale; CI, confidence interval; PSS, Perceived Stress Scale; SEP, socioeconomic position; WHO, World Health Organization.

Appendix S1 Description of the Survey Questionnaire

แบบวัดความรู้สึกเครียด (T-PSS-10)

คำชี้แจง ต่อไปนี้เป็นคำถามเกี่ยวกับความรู้สึกและความคิดของท่านในรอบ 1 เดือนที่ผ่านมา กรุณาเลือกคำตอบที่ตรงกับที่ท่านคิดหรือรู้สึกแบบนั้นมากที่สุด

**พฤติกรรมและการกระทำที่ตรงกับท่าน**		**ความรู้สึกที่ตรงกับท่านมากที่สุด**				
		0 ไม่เลย	1 แทบจะไม่มี	2 มีบางครั้ง	3 ค่อนข้างบ่อย	4 บ่อยมาก
1.	ในรอบ 1 เดือนที่ผ่านมา บ่อยแค่ไหนที่ท่าน รู้สึกไม่สบายใจเพราะมีสิ่งที่เกิดขึ้นอย่างไม่คาดคิด	☐	☐	☐	☐	☐
2.	ในรอบ 1 เดือนที่ผ่านมา บ่อยแค่ไหนที่ท่าน รู้สึกว่าท่านไม่สามารถควบคุมเรื่องสำคัญ ๆ ในชีวิตของท่านได้	☐	☐	☐	☐	☐
3.	ในรอบ 1 เดือนที่ผ่านมา บ่อยแค่ไหนที่ท่าน รู้สึกกระสับกระส่ายและตึงเครียด	☐	☐	☐	☐	☐
4.	ในรอบ 1 เดือนที่ผ่านมา บ่อยแค่ไหนที่ท่าน รู้สึกมั่นใจในความสามารถของตนเองที่จะรับมือกับปัญหาส่วนตัวทั้งหลายได้	☐	☐	☐	☐	☐
5.	ในรอบ 1 เดือนที่ผ่านมา บ่อยแค่ไหนที่ท่าน รู้สึกว่าสิ่งทั้งหลายเป็นไปในทิศทางที่ท่านต้องการ	☐	☐	☐	☐	☐
6.	ในรอบ 1 เดือนที่ผ่านมา บ่อยแค่ไหนที่ท่าน รู้สึกว่าท่านไม่สามารถจัดการกับสิ่งทั้งหลายทั้งที่เป็นสิ่งที่ล้วนเคยทำมาแล้ว	☐	☐	☐	☐	☐
7.	ในรอบ 1 เดือนที่ผ่านมา บ่อยแค่ไหนที่ท่าน รู้สึกว่าสามารถควบคุมสิ่งทั้งหลายที่มากวนใจได้	☐	☐	☐	☐	☐
8.	ในรอบ 1 เดือนที่ผ่านมา บ่อยแค่ไหนที่ท่าน รู้สึกว่าท่านควบคุมสถานการณ์ต่าง ๆ ได้	☐	☐	☐	☐	☐
9.	ในรอบ 1 เดือนที่ผ่านมา บ่อยแค่ไหนที่ท่าน โกรธอันเนื่องมาจากสิ่งที่นอกเหนือการควบคุมของท่าน	☐	☐	☐	☐	☐
10.	ในรอบ 1 เดือนที่ผ่านมา บ่อยแค่ไหนที่ท่าน รู้สึกว่าปัญหาต่าง ๆ ทับถมมากขึ้นจนท่านไม่สามารถแก้ไขได้หมด	☐	☐	☐	☐	☐

แบบประเมินความสามารถในการปรับตัวและเผชิญกับปัญหา (BRCS)

คำชี้แจง ให้ท่านพิจารณาว่าข้อความต่อไปนี้ อธิบายพฤติกรรมและการกระทำของท่านได้ดีมากน้อยเพียงใด โดยทำเครื่องหมาย ✓ ลงในช่อง ที่ตรงกับพฤติกรรมและการกระทำของท่านมากที่สุด

พฤติกรรมและการกระทำที่ตรงกับท่านมากที่สุด	1	หมายถึง	ไม่เลย
	2	หมายถึง	แทบจะไม่เลย
	3	หมายถึง	ตรงกับตัวฉันบ้าง
	4	หมายถึง	ค่อนข้างตรงกับตัวฉัน
	5	หมายถึง	ตรงกับตัวฉันมากที่สุด

**พฤติกรรมและการกระทำที่ตรงกับท่าน**	1	2	3	4	5
1. ฉันมองหาวิธีการใหม่ ๆ ที่ดี ในการจัดการกับสถานการณ์ที่ยากลำบาก	☐	☐	☐	☐	☐
2. ไม่ว่าจะเกิดอะไรขึ้นกับฉัน ฉันเชื่อว่าฉันสามารถควบคุมการกระทำของฉันได้	☐	☐	☐	☐	☐
3. ฉันเชื่อว่าฉันสามารถเรียนรู้ไปในทิศทางที่ดีขึ้นได้ เมื่อเผชิญกับสถานการณ์ที่ยากลำบาก	☐	☐	☐	☐	☐
4. ฉันพยายามหาวิธีที่จะทดแทนความสูญเสียที่ฉันได้ประสบในชีวิต	☐	☐	☐	☐	☐

ดัชนีชี้วัดสุขภาวะขององค์การอนามัยโลก (WHO-5)

คำชี้แจง กรุณาตอบข้อคำถามในแต่ละข้อต่อไปนี้ว่าท่านมีความรู้สึกต่อข้อคำถามนั้น มากน้อยเพียงใด โดยใช้เครื่องหมาย ✓ ลงในช่อง ที่ตรงกับความรู้สึกเกิดขึ้นกับท่านมากที่สุด

ความรู้สึกที่ตรงกับท่านมากที่สุด	0	หมายถึง	ไม่เคยเลย
	1	หมายถึง	มีบ้าง บางเวลา
	2	หมายถึง	น้อยกว่าครึ่งหนึ่ง
	3	หมายถึง	มากกว่าครึ่งหนึ่ง
	4	หมายถึง	เป็นส่วนใหญ่
	5	หมายถึง	ตลอดเวลา

**ภายใน 2 สัปดาห์ที่ผ่านมา**	**ความรู้สึกที่ตรงกับท่านมากที่สุด**					
	0	1	2	3	4	5
1. ฉันรู้สึกเบิกบานและอารมณ์ดี	☐	☐	☐	☐	☐	☐
2. ฉันรู้สึกสงบและผ่อนคลาย	☐	☐	☐	☐	☐	☐
3. ฉันรู้สึกกระฉับกระเฉงและมีพลัง	☐	☐	☐	☐	☐	☐
4. ฉันตื่นขึ้นมาด้วยความรู้สึกสดชื่นและได้พักเพียงพอ	☐	☐	☐	☐	☐	☐
5. ชีวิตประจำวันของฉันเต็มไปด้วยสิ่งที่น่าสนใจ	☐	☐	☐	☐	☐	☐

ข้อมูลทั่วไปของผู้ตอบแบบสอบถาม

1. อายุ .... (ปี)
2. เพศ
   - ☐ ชาย
   - ☐ หญิง
   - ☐ อื่น ๆ (ไม่ประสงค์ระบุ)
   - ☐ อื่น ๆ โปรดระบุ ....
3. สถานภาพการสมรส
   - ☐ โสด
   - ☐ สมรส
   - ☐ หม้าย/หย่าร้าง
   - ☐ อื่น ๆ โปรดระบุ ....
4. ศาสนา
   - ☐ ไม่มี
   - ☐ พุทธ
   - ☐ คริสต์
   - ☐ อิสลาม
   - ☐ อื่น ๆ โปรดระบุ ....
5. อาศัย/ทำงานอยู่ในภูมิภาค
   - ☐ กรุงเทพมหานครและปริมณฑล
   - ☐ ภาคเหนือ
   - ☐ ภาคกลาง
   - ☐ ภาคตะวันออกเฉียงเหนือ
   - ☐ ภาคตะวันตก
   - ☐ ภาคตะวันออก
   - ☐ ภาคใต้
6. ลักษณะการพักอาศัย
   - ☐ พักอาศัยอยู่คนเดียว
   - ☐ พักอาศัยกับครอบครัว
   - ☐ พักอาศัยกับผู้อื่น ที่มิใช่บุคคลในครอบครัว
7. จำนวนสมาชิกที่พักอาศัยในบ้านหรือห้องเช่าเดียวกัน (รวมท่านด้วย) .... (คน)

8. ท่านมีห้องนอนส่วนตัวหรือไม่

☐ ไม่มี (ใช้ร่วมกับผู้อื่น ที่มิใช่คู่สมรส)

☐ มีส่วนตัวสำหรับตนเอง

☐ มี ใช้ร่วมกับคู่สมรส

9. จำนวนห้องนอน ทั้งหมดในบ้านหรือห้องเช่า .... (ห้อง)

10. รายได้ของท่านต่อเดือน .... (บาท)

11. แหล่งที่มาของรายได้

☐ ตนเอง

☐ ผู้อื่น

☐ ตนเองและผู้อื่น

12. ท่านสูญเสียเสียรายได้จากสถานการณ์การระบาดของเชื้อไวรัสโควิด-19 (COVID-19) หรือไม่

☐ ไม่ใช่

☐ ใช่ กรุณาระบุ .... (บาท/เดือน)

13. ท่านมีปัญหาทางการเงิน หรือรายได้ไม่เพียงพอจากสถานการณ์การระบาดของเชื้อไวรัสโควิด-19 (COVID-19) หรือไม่

☐ รายได้ไม่เพียงพอ

☐ รายได้เพียงพอ

14. ณ ขณะนี้ ท่านมีหนี้สินหรือไม่

☐ ไม่มี

☐ มีหนี้สินในระบบ

☐ มีหนี้สินนอกระบบ

☐ มีหนี้สินทั้งในและนอกระบบ

15. สิทธิ์การรักษาพยาบาล

☐ บัตรประกันสุขภาพถ้วนหน้า (บัตรทอง 30 บาท)

☐ ข้าราชการ

☐ รัฐวิสากิจ

☐ ประกันสังคม

☐ จ่ายเอง

☐ อื่น ๆ โปรดระบุ ....

16. ท่านเคยได้รับการวินิจฉัยว่ามีปัญหาทางสุขภาพจิตมาก่อนหรือไม่

☐ ไม่เคย

☐ เคย

17. ณ ขณะนี้ท่านกำลังรับบริการการรักษาทางสุขภาพจิตหรือไม่

☐ ไม่ใช่

☐ ใช่

18. โรคประจำตัว **(เลือกได้หลายคำตอบ)**

☐ ไม่มี

☐ เบาหวาน

☐ ความดันโลหิตสูง

☐ ไขมันในเลือดสูง

☐ โรคหัวใจ

☐ โรคไตเรื้อรัง

☐ อื่น ๆ โปรดระบุ ....

19. ระดับการศึกษา

☐ ไม่ได้เรียนหนังสือ

☐ ประถมศึกษา

☐ มัธยมศึกษาตอนต้น

☐ มัธยมศึกษาตอนปลาย/ปวช

☐ อนุปริญญา/ปวส

☐ ปริญญาตรี

☐ สูงกว่าปริญญาตรีหรือเทียบเท่า

☐ อื่น ๆ โปรดระบุ ....

20. อาชีพ

☐ ว่างงาน/ตกงาน

☐ เกษียณอายุ/ไม่ได้ประกอบอาชีพ

☐ เกษตรกร

☐ รับจ้าง

☐ ค้าขาย

☐ ข้าราชการ

☐ พนักงานราชการ

☐ รัฐวิสาหกิจ

☐ อาชีพอิสระ (freelance)

☐ อื่น ๆ โปรดระบุ ....
